# Transcriptomic Signatures of Airway Epithelium Infected With SARS-CoV-2: A Balance Between Anti-infection and Virus Load

**DOI:** 10.3389/fcell.2021.735307

**Published:** 2021-08-23

**Authors:** Lingzhang Meng, Houji Qin, Jingjie Zhao, Siyuan He, Qiuju Wei, Zechen Wang, Jiajia Shen, Suren Sooranna, Jian Song

**Affiliations:** ^1^Center for Systemic Inflammation Research (CSIR), School of Preclinical Medicine, Youjiang Medical University for Nationalities, Baise, China; ^2^Department of Infectious Diseases, Affiliated Hospital of Youjiang Medical University for Nationalities, Baise, China; ^3^Life Science and Clinical Research Center, Affiliated Hospital of Youjiang Medical University for Nationalities, Baise, China; ^4^College of Pharmacy, Youjiang Medical University for Nationalities, Baise, China; ^5^Department of Metabolism, Digestion and Reproduction, Imperial College London, Chelsea and Westminster Hospital, London, United Kingdom; ^6^Department of Radiation Oncology, Renji Hospital, School of Medicine, Shanghai Jiao Tong University, Shanghai, China

**Keywords:** COVID-19, ARDS, scRNA seq, viral infection, innate immunity

## Abstract

COVID-19 pneumonia requires effective medical therapies. However, it is a challenge to find therapeutic drugs that not only inhibit viral replication, but also inhibit the accompanying cytokine storm and maintain an appropriate immune response. In this study, the effects of SARS-CoV-2 on gene expression in lung epithelial cells from patients with COVID-19 were systematically evaluated with bioinformatics analysis methods. Transcriptome expression specific to bystander (exposed but uninfected) and infected cells were found, and the vital pathways were identified by conducting differentially expressed gene analysis regarding the relationship between gene signatures of COVID-19 infection and disease severity. We found that a high viral load did not necessarily imply a low response of epithelial cells or a poor disease convalescence. The ability to distinguish the role of virus-correlated genes facilitates the development of potential new medicines and therapies for COVID-19 infection.

## Introduction

Coronavirus disease 2019 (COVID-19) is confirmed as an acute respiratory infectious disease that has been attributed to infection with the novel severe acute respiratory syndrome coronavirus 2 (SARS-CoV-2) (Huang et al., 2020). Only a few effective therapeutic agents have been developed to combat the transmissibility and infectivity of SARS-CoV-2. Although the global medical community is currently conducting large-scale clinical trials to verify whether any of the existing drugs are effective in treating COVID-19 patients, a systematic analysis method is required to detect the target genes for any medical treatment (Li G. et al., 2021).

When coronaviruses enter lung epithelial cells, type I interferons in these cells are activated, thereby preventing the virus from replicating within the cell. How to target viral replication without affecting the interferon pathway turns out to be the toughest problem in drug therapy. Given that the production of type I interferon in the lung epithelial cells is relatively low, the replication and expansion of the virus is not limited, thereby allowing the release of progeny viruses, causing cell anxiety and stress, and then activating the innate and adaptive immune systems in the body (Acharya et al., 2020; King and Sprent, 2021). On that basis, considerable various inflammatory cytokines and chemokines are produced, and monocytes as well as B and T cells are attracted to the site of infection. This establishes the formation of a feedback loop, and then more inflammatory cytokines are synthesized, which regulates and diminishes the viral infection. However, a dysfunctional immune response attracts excessive immune cells to reach the lung tissue, thereby leading to the excessive production and secretion of inflammatory cytokines as well as a cytokine storm, i.e., an important factor that can lead to acute respiratory distress syndrome (ARDS) (Fajgenbaum and June, 2020). ARDS is the main cause of death as a result of novel coronavirus pneumonia. Therefore, an ideal treatment should inhibit the reproduction or replication of the virus that causes COVID-19, and antiviral immunity responses should be maintained to an adequate level. To achieve this, a precise understanding of the relationships between virus infection and disease severity is needed.

This study aimed to systematically assess the effects of SARS-CoV-2 on gene expression in the epithelial as well as various immune cells in the lung tissue of COVID-19 patients. The differences seen between COVID-19 pneumonia patients were compared with those with bacterial pneumonia and healthy subjects. This allowed the identification of the transcriptome expression specific to bystander (exposed but uninfected) and infected cells as well as important genes related to ARDS severity by conducting differentially expressed gene (DEG) analysis. It is envisaged that this will provide insights into the balance between anti-infection and the prevention of excessive immune responses in patients with COVID-19.

## Materials and Methods

### Single Cell Sequencing Analysis of Lung Lavage Cells From COVID-19 Patients

The scRNA-seq data of human bronchoalveolar lavage fluid (BALF) originated from the gene expression omnibus database and were accessed through NCBI GSE155249 (Grant et al., 2021), GSE167118 (Zhao M.M. et al., 2021), and GSE157526 (Fiege et al., 2021) as well as the raw data from Lambrechts’ laboratory (Wauters et al., 2021). GSE155249 was used for the *in vivo* study of COVID-19–related genes and the subsequent gene signature. GSE157526 was used for the *in vitro* study of COVID-19–related genes and the subsequent gene signature. Metadata from GSE167118 and Lambrechts (Wauters et al., 2021) were used to classify the genes into severe or mild ARDS groups. The data set GSE155249 comprised 10 samples infected with coronavirus isolated from patients during COVID-19 pneumonia, one sample with bacterial pneumonia, and one normal human biopsy sample without a history of pneumonia-related disease, which was thereby expressed as the non-pneumonia group. In the present study, cells with at least 200 genes as well as genes expressed in at least three cells were retained during analysis. The information-rich resource (Ren et al., 2021) see text footnote 5 was used for the analysis of correlations of the target genes with disease progression or convalescence.

### Single-Cell RNA-Seq Data Analysis

Specific to the integrated analysis of single-cell data, the data from infected new coronavirus and bacterial pneumonia samples as well as non-pneumonia samples were normalized using the SCTransform method (Stuart et al., 2019). These were then analyzed by conducting mutual principal component analysis (PCA)^1^. The PCA analysis was further conducted for the integrated data sets, and cluster analysis was performed with uniform manifold approximation and projection (UMAP). The cluster analysis of single-cell data was performed with Seurat’s graph-based clustering method. The resolution of the FindClusters feature was set to 0.1. Subsequently, the clusters were visualized with the UMAP version 0.2.6.0 graph. The R software package Seurat (version 2.3.4) was used for the data analysis. During quality control, unique molecular modifier (UMI) counts of less than 500 and those with double multiples were removed. Furthermore, cells with >5% of mitochondrial genes and >50% of ribosomal genes were filtered out.

### Genetic Characterization of Lung Tissue From COVID-19 Patients

The cells were first sorted into 10 cell types by complying with the single-cell data, and they were classified as samples infected with the new coronavirus and bacterial pneumonia as well as non-pneumonia samples. They were then divided into either a virus-infected lung-infection cell group or a virus-infected group and were further subdivided into high- and low-infection groups. The corresponding transcriptome analysis data were compared to screen DEGs, and to increase the efficiency of the study, among the mentioned candidate DEGs and the parameters used were min.pct > 0.25 and | Log2 (FC) | > 0.5. The DEGs underwent pathway enrichment analysis, and their gene expression profiles were analyzed using the Gene Set Enrichment Analysis (GSEA) method together with data sets from the MSigDB database (Meng et al., 2021; Xing and Song, 2021).

### GSEA Method for the Enrichment Analysis of GO Ontology and KEGG Pathway

The gene features were processed and then analyzed with the method at WebGestalt webserver for the annotation of involved GO ontology and KEGG pathway (Sun et al., 2018).

### Extraction Method of Intersection Element

The DEG data were read with the VennDiagram package (Chen and Boutros, 2011), and the intersection status between the groups was counted. Moreover, after the information was acquired on the number of consensus elements, a Venn diagram was drawn. The intersection data were extracted using the VennDetail package^2^. The differential output and comparison of the relevant genes of the mentioned intersection elements were also exploited to map with the corrplot software package^3^ or output for signaling pathway enrichment analysis based on the use of GSEA (Li R. et al., 2021).

### DEG Correlation Analysis in Expression Matrix of Each Group

Specific to the one-to-one correlation analysis, the FeatureScatter function in Seurat was applied. For many-to-many correlation analysis, the single-cell expression matrix after grouping or after cell type was read with Seurat and integrated with the DEG list of the respective group so as to obtain the single-cell expression matrix of DEGs. Then, the correlation coefficient between DEGs and viral gene expression was calculated and plotted using corrplot.

### Protein–Protein Interaction

The STRING database^4^ was employed to build up the protein–protein interaction (PPI) network. Cytoscape was used to demonstrate the node and edge of the PPI network. CytoHubba was used to screen the hub genes, and the top 20 genes that were ranked by degree are shown.

## Results

**FIGURE 1:**
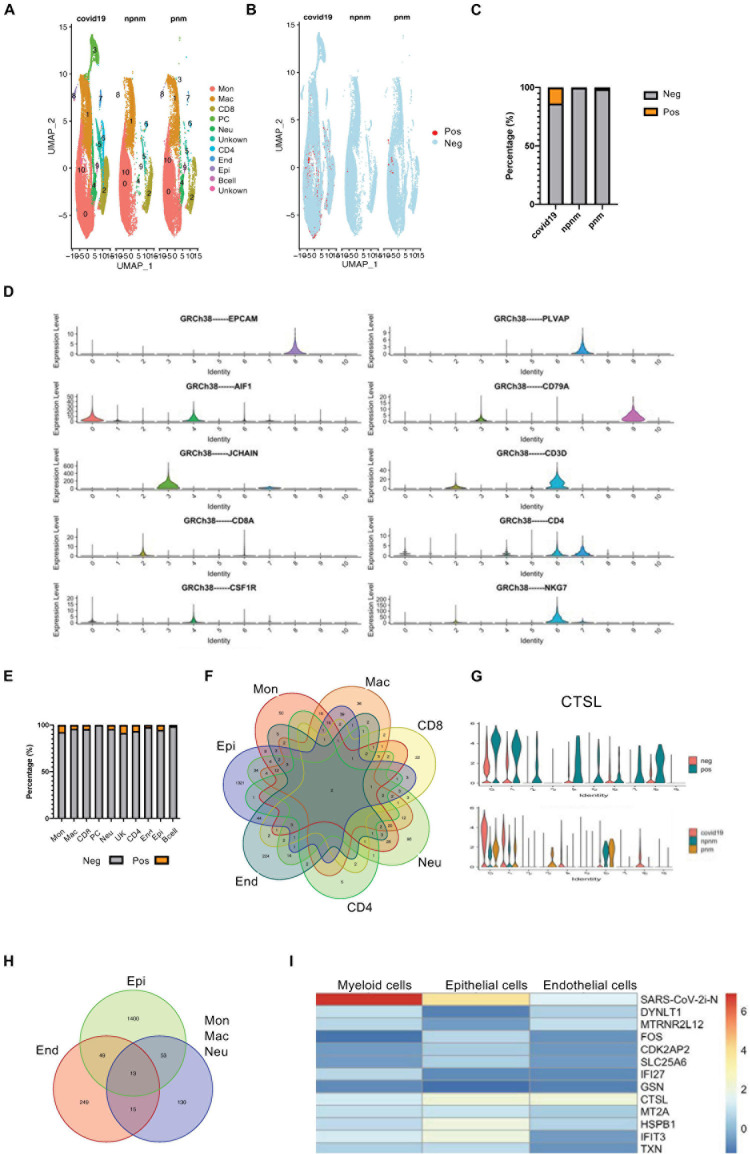
Transcriptome profiling of SARS-CoV-2–infected cells. **(A)** A UMAP diagram showing the cell clusters in COVID-19-related pneumonia (covid19), control (npnm), and bacterial pneumonia (pnm) samples. **(B)** A UMAP diagram showing the SARS-CoV-2 positive and negative cells in the COVID-19 pneumonia (covid19), control (npnm), and bacterial pneumonia (pnm) samples. **(C)** Bar charts to quantify and compare the proportion of SARS-CoV-2 positive and negative cells in the COVID-19 pneumonia (covid19), control (npnm), and bacterial pneumonia (pnm) samples. **(D)** Violin plots indicating the expression and distribution of marker genes in monocytes (c0), macrophages (c1), CD8 + T cells (c2), plasma cells (c3), neutrophils (c4), CD4 + T cells (c6), endothelial cells (c7), epithelial cells (c8), and B cells (c9). **(E)** Bar charts to quantify and compare the proportion of SARS-CoV-2 positive and negative cells in the main cell types. **(F)** A Venn diagram highlighting the intersecting gene signatures of SARS-CoV-2–infected ells. **(G)** Violin plots indicating the levels of CTSL expression in the SARS-CoV-2-infected and non-infected main cell types. Violin plots indicating the levels of CTSL expression in the SARS-CoV-2-infected and non-infected cell populations in COVID-19 pneumonia (covid19), control (npnm) and bacterial pneumonia (pnm) samples. **(H)** A Venn diagram highlighting the intersecting gene signatures of SARS-CoV-2–infected myeloid, endothelial, and epithelial cells. **(I)** A heat map revealing the fold changes of the shared DEGs in the COVID-19-related myeloid, epithelial, and endothelial cells.

BALF single-cell RNA sequencing data was used to study SARS-CoV-2 infection responses *in vivo* to study the transcriptome of different cells and compare non-COVID-19 patients with bacterial pneumonia or healthy subjects. In addition, the DEG transcriptomes of bystander (exposed but uninfected) and infected cells (COVID-19 gene-expressing cells) (Figures 1A–C and Supplementary Figures 1A–C) were also explored. During data mining, human BALF cells were classified into 10 clusters with plasma cells appearing only in COVID-19–infected patients, and the main cell types were monocytes, macrophages, T cells and neutrophils, endothelial cells, and epithelial cells (Figures 1A,D). Using the expression of COVID-19–related viral genes, the infected cells could be separated from uninfected cells, and the viral genes in the infected cells were mainly N, S, and M genes (Figure 1C and Supplementary Figure 1D). As indicated from the analysis, there was a significant level of viral gene expression in monocytes, neutrophils, macrophages, and T cells as well as the predicted epithelial cells although their roles in the different cell types require further in-depth studies (Figure 1E and Supplementary Figure 1D).

Although the proportion of SARS-CoV-2-positive cells in BALF was relatively similar (Figure 1E and Supplementary Figure 1D), we found only two consensus genes from different cell types—one for the N gene of the virus and the other for the CTSL gene (Figure 1F)—when we compared the differential gene expression in COVID-19-positive to -negative cells. The upregulation of CTSL in all cells was consistent with the fact that CTSL promotes SARS-CoV-2 infection (Zhao Y. et al., 2021). When COVID-19 pneumonia patients and bacterial pneumonia or healthy controls were compared, CTSL was upregulated in monocytes, macrophages, neutrophils, and epithelial cells in COVID-19 pneumonia patients. However, CTSL tended to be comparable in CD4 + T cells from COVID-19 pneumonia patients, suggesting the particularity of CTSL in T cells infected with the virus that causes COVID-19 (Figure 1G). Concerning the similarity, myeloid cells were grouped and compared with epithelial and endothelial cells for the SARS-CoV-2-positive cell upregulated genes, resulting in 13 consensus genes (Figure 1H and Supplementary Figures 2A,B). When comparing the expression variations of these 13 consensus genes in myeloid, epithelial, and endothelial cells, the myeloid cells were suggested to have stronger upregulation of N gene expression relative to the other two cell types. However, the CTSL gene, the IFN-inducible gene IFIT3, and the IFN-repressed gene HSPB1 were more strongly upregulated in the epithelial cells, which reflected the activation of innate immune signaling pathways in these cells (Figure 1I; Hachim et al., 2020).

**FIGURE 2:**
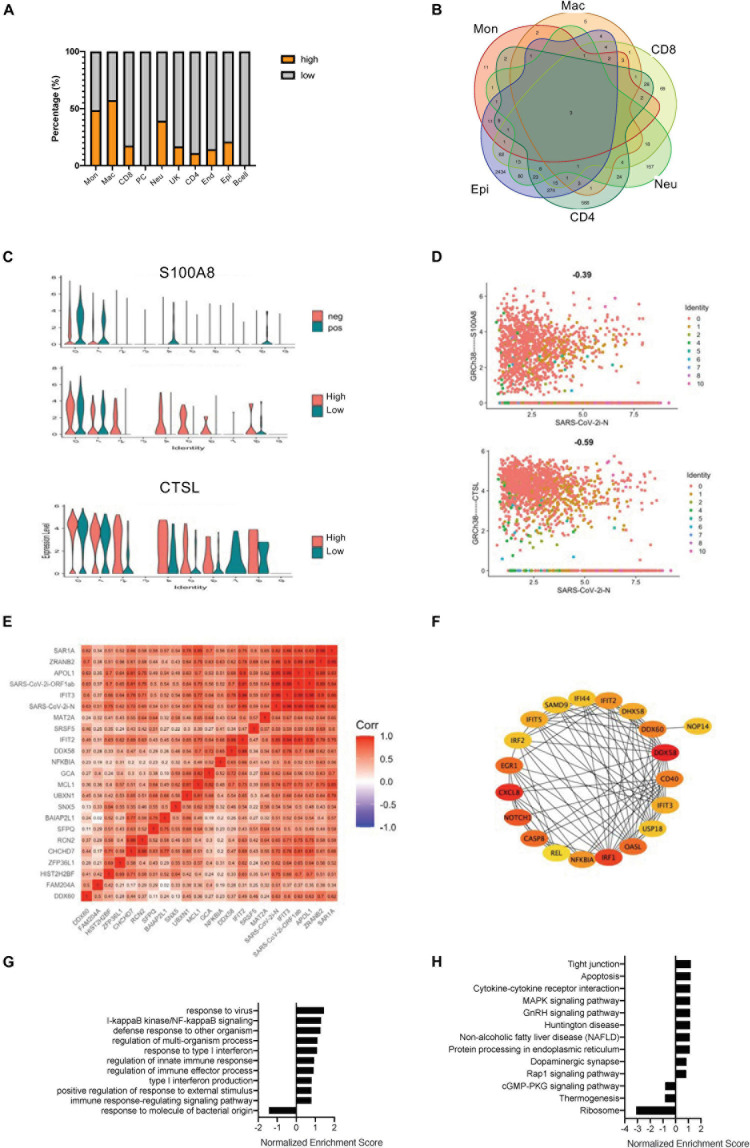
Correlation of SARS-CoV-2 infection with the COVID-19–related gene signatures. **(A)** Bar charts to quantify the proportion of heavily loaded SARS-CoV-2 in the main cell types. **(B)** A Venn diagram highlighting the intersecting gene signatures of heavily loaded SARS-CoV-2 cells. **(C)** Violin plots indicating the levels of S100A8 expression in SARS-CoV-2 positive and negative cells. Violin plots indicating the expression levels of S100A8 and CTSL in heavily loaded SARS-CoV-2 cells. **(D)** Feature plots revealing the relationship between COVID-19–related genes and either S100A8 or CTSL gene expression in heavily loaded SARS-CoV-2 cells. **(E)** A heat map revealing the relationship between COVID-19–related genes and DEGs in the SARS-CoV-2-infected epithelial cells. **(F)** The PPI network demonstrating the COVID-19–related infection-correlated hub gene signatures in the SARS-CoV-2-infected epithelial cells. **(G)** The enriched gene ontology of GSEA analysis for SARS-CoV-2-infected epithelial cell-correlated gene signatures. **(H)** The enriched KEGG signaling pathway of GSEA analyses for SARS-CoV-2-infected epithelial cell-correlated gene signatures.

In a study of SARS-CoV-2-infected cells, it was found that, although the viral infection rate of each cell was relatively consistent, the amount of infection could be very different (Supplementary Figure 1D). All cells expressing the N gene of COVID-19 greater than five copies were further defined as the highly infected cells (Supplementary Figures 3A,B). The main highly infected cell types included monocytes, macrophages, neutrophils, and epithelial cells (Figure 2A and Supplementary Figure 3B). Although endothelial cells had some viral infections, further studies could not be pursued due to the lack of enough cells. Moreover, the gene expression differences between the highly infected cells and the less infected cells were investigated, and the DEGs of all highly infected cell types were pooled. Only three intersection genes were obtained, two of which were COVID-19 viral genes, and the other was a DAMP molecule, S100A8 (Chen et al., 2020; Figures 2B–D). S100A8 and CTSL were upregulated in similar cell types except for monocytes and macrophages (Figure 2C). The grouping of high and low infections did not reflect the relationship of gene expression with COVID-19 virus infection in a single cell. With scatterplots, the relationship between S100A8 and CTSL genes and SARS-CoV-2 infection was suggested to be only negatively related to viral gene expression levels in high infections, and all COVID-19–related viral genes, especially N and ORF1ab genes, showed a positive relationship (Figure 2D and Supplementary Figures 4A,B).

To more effectively study the relationship between the degree of viral infection and the gene signatures, a series of comprehensive correlation studies were performed. In monocytes, macrophages and neutrophils, viral expressed genes showed high correlation only with virally expressed genes (VEGs), but significantly low correlation with other genes (Supplementary Figures 4C,D,F). In CD8 + T cells, VEGs were negatively related to the NF-κB gene and also with the IFN-related gene, IRF1 (Supplementary Figure 4E). In CD4 + T cells, a considerable number of genes were positively related to SARS-CoV-2 infection, including genes related to cell motility (ITGA6 and MUC1) and genes related to cell activation (such as IL7R) (Supplementary Figure 4G). The largest number of gene associations was also found in the epithelial cells, which is consistent with previous genetic studies of SARS-CoV-2-positive cells (Figure 2E). We further performed gene enrichment analysis and constructed a PPI network based on the genes in the epithelial cells of the highly infected groups (Figure 2F and Supplementary Figure 4H). Gene ontology enrichment revealed that most of the hub genes were related to NF-κB and interferon-induced signaling (Figure 2G). KEGG signaling studies suggested that the DEGs in the epithelial cells of the highly infected group mainly clustered in cytokines and cytokine interactions (Figure 2H). An interesting phenomenon here is that the SARS-CoV-2–infected epithelial cells were very sensitive to viral replication, which correlated significantly with genes related to early defense mechanisms.

**FIGURE 3:**
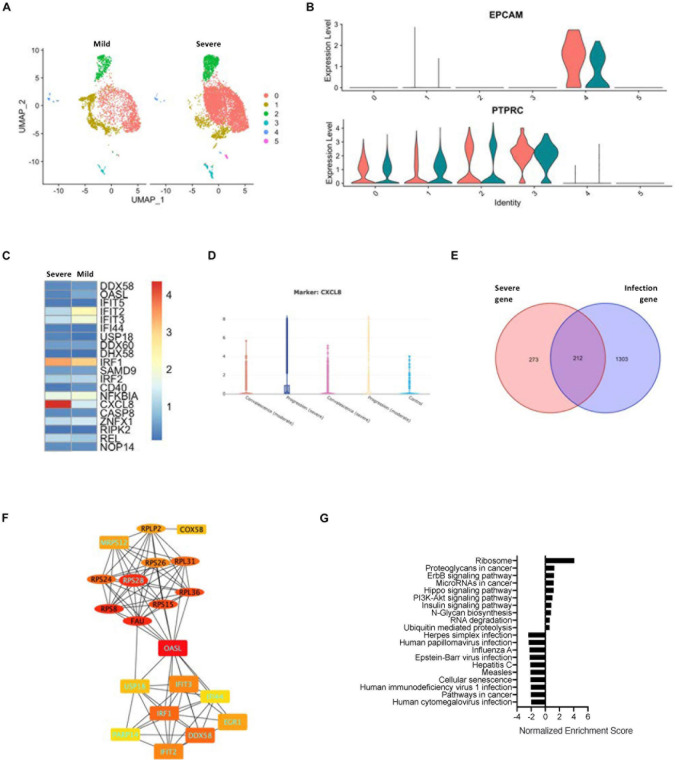
Relationship between SARS-CoV-2 infection in the epithelial cells and severity of COVID-19. **(A)** A UMAP diagram showing an overlay of the main cell types of the severe and mild ARDS BALF samples. **(B)** Violin plots to annotate the leukocytes and epithelial cells in the severe and mild ARDS BALF samples. **(C)** A heat map demonstrating the expression levels of the COVID-19-related PPI hub genes in the severe and mild ARDS BALF samples. **(D)** Violin plots showing the expression of CXCL8 in the severe disease progression group. **(E)** A Venn diagram highlighting the intersection between COVID-19–related infection-correlated genes and severe ARDS-related genes. **(F)** The PPI network demonstrating the shared hub gene signatures between COVID-19–related infection-correlated genes and the severe ARDS-related genes. The oval structures indicate the upregulated genes, and the rectangles indicate the downregulated genes in the severe ARDS cases. Genes labeled with light blue text are positively correlated with SARS-CoV-2 infection. **(G)** A GSEA summary plot of enriched KEGG signaling for the shared gene signature of COVID-19–related infection-correlated genes and the severe ARDS-related genes.

As SARS-CoV-2 infection-correlated genes cannot reflect the risk factors, we used the data set that comprised both gene expression and disease severity (Figure 3A). Although the epithelial cells from the COVID-19 patients with either severe or mild symptoms exhibited a similar level of EPCAM gene expression (Figure 3B), the hub gene expression was clearly altered between the two groups of patients. The levels of CXCL8 were enhanced drastically in the severe group (Figure 3C and Supplementary Figure 5A). Taking advantage of the web browser of another COVID-19 scRNA resource^5^, we were able to classify the upregulation of CXCL8 in the progression group from the patients with severe ARDS (Figure 3D). Furthermore, we compared the epithelial cell signature genes between the COVID-19 infection-related genes and the severe ARDS-related genes and found that a total of 212 intersection genes were present in both situations (Figure 3E). A PPI network analysis of the intersect genes revealed two central hub gene connections: one for the interferon-related genes that are downregulated in severe ARDS and the other for the ribosome-related genes that are upregulated in the severe ARDS patients (Figure 3F and Supplementary Figure 5B). Then, gene enrichment analysis was conducted for intersecting genes for the infected epithelial cells of severe ARDS cases, and it demonstrated 35 corresponding up- or downregulated KEGG signaling pathways (Figure 3G).

**FIGURE 4:**
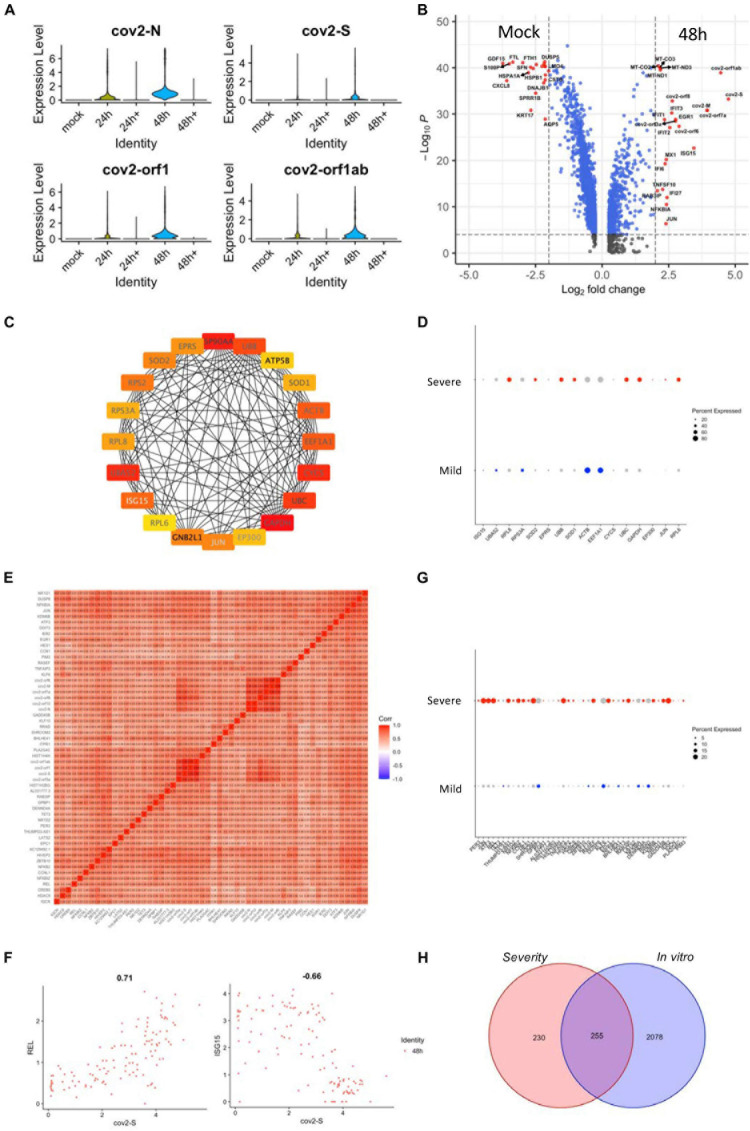
Gene expression analyses of SARS-CoV-2-infected epithelial cell lines. **(A)** Violin plots demonstrating the expression patterns and levels of the COVID-19-related genes in SARS-CoV-2-infected and non-infected epithelial cell lines. Cultured cells were inoculated with SARS-CoV-2 for 24 and 48 h. 24 h + and 48 h + indicate co-culture with Remdesivir for the respective time periods. **(B)** A volcano plot demonstrating the fold change and the significance of the DEGs of infected and non-infected (mock) epithelial cells. **(C)** The PPI network demonstrating the hub gene signatures in SARS-CoV-2–infected epithelial cell lines. **(D)** Dot plots demonstrating the expression of hub genes in the severe and mild ARDS BALF epithelial cells. **(E)** A heat map revealing the relationship between COVID-19–related genes and SARS-CoV-2–infected epithelial cell line–related DEGs. **(F)** Feature plots revealing the relationship between COVID-19–related genes and either REL or ISG15 gene expression in SARS-CoV-2–infected epithelial cell lines. **(G)** Dot plots demonstrating the expression of COVID-19-related correlated genes in the severe and mild ARDS BALF epithelial cells. **(H)** A Venn diagram demonstrating the intersection between SARS-CoV-2-infected epithelial cell line–related genes and severe ARDS-related genes.

The transcriptional profile of epithelial cells may exhibit significant complexity and heterogeneity due to the antiviral response of different cell types *in vivo*. Therefore, we analyzed the transcriptional profile of normal human tracheal bronchial epithelial (nHTBE) cells infected with SARS-CoV-2 to investigate the self-response of epithelial cells (Fiege et al., 2021). Cultured cells inoculated with SARS-CoV-2 showed 100% infection in the cells after 48 h incubation (Figure 4A). We found that 48 h postinfection showed an upregulation of the hub genes using the *in vivo* scRNA assay (Supplementary Figure 6A), suggesting this time point could be used to compare with the uninfected cells and the *in vivo* data. As expected, the SARS-CoV-2–infected epithelial cells showed marked upregulation of interferon-induced genes (ISGs) such as ISG15, IFIT3, and MX1, compared to non-infected (mock) epithelial cells (Figure 4B), and this was also revealed by the PPI network analysis as one of the hub genes (Figure 4C). The expression analysis in the severity group indicated that ISG15 should belong to the mild group, matching the protective role of the ISGs from SARS-CoV-2 infection (Figure 4D). Also, we performed a correlation study with *in vitro* infected cell-related genes, which showed a correlation of SARS-CoV-2 infection with the NF-κB family of transcription factors, such as REL, and a negative correlation with ISG15 (Figures 4E,F and Supplementary Figure 6B). The REL expression was correlated with the severe group signature genes (Figure 4G). In total, there are 255 intersective genes between the severity-related genes and the *in vitro* infection-related genes (Figure 4H), which accounts for more than half of the severity-related genes and suggests the importance of the *in vitro* results.

**FIGURE 5:**
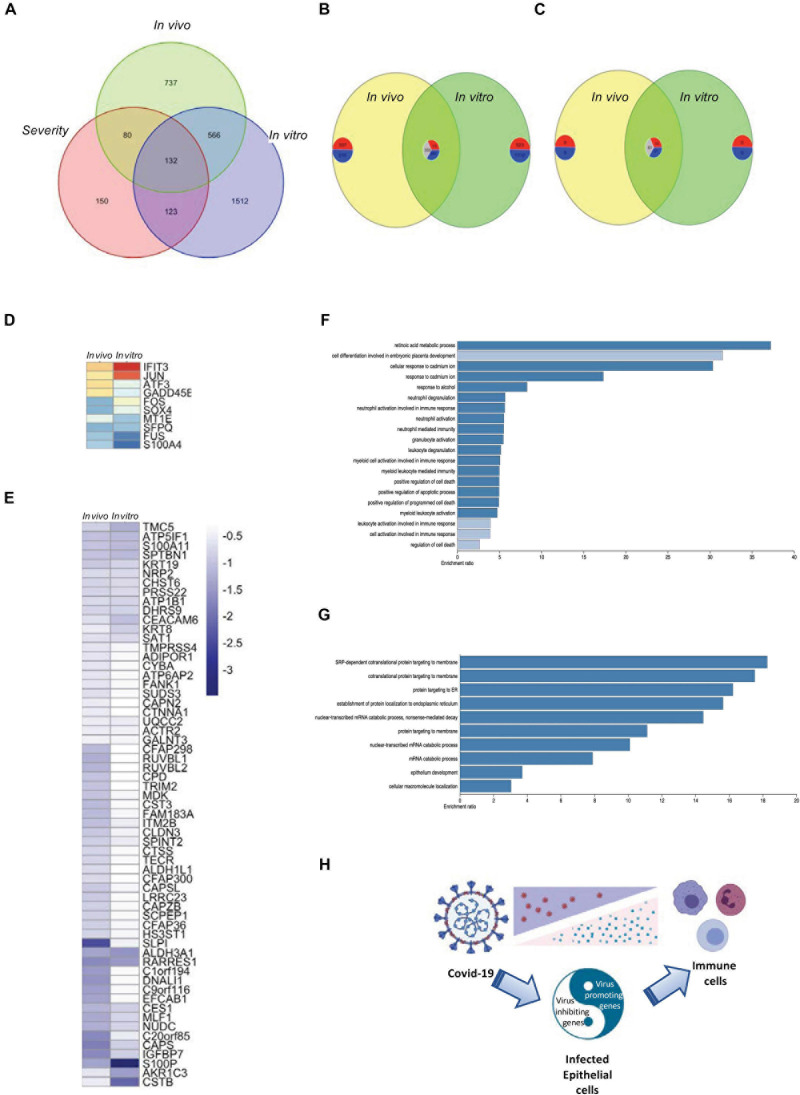
Overlapping of *in vivo* and *in vitro* gene expression and disease severity. **(A)** A Venn diagram demonstrating the intersection of *in vivo* and *in vitro* SARS-CoV-2 infection-related genes and severe ARDS-related genes. **(B)** A Venn diagram demonstrating the shared genes of *in vivo* and *in vitro* SARS-CoV-2 infection–related genes. The number of commonly upregulated, commonly downregulated, and contra-regulated genes are shown in a clockwise manner. **(C)** A Venn diagram demonstrating the shared genes of *in vivo* and *in vitro* SARS-CoV-2 infection–related genes with severe ARDS-related genes. The number of commonly upregulated, commonly downregulated, and contra-regulated genes are shown in a clockwise manner. **(D)** A heat map demonstrating the expression levels of the commonly upregulated genes of *in vivo* and *in vitro* SARS-CoV-2 infection–related genes that were shared with severe ARDS-related genes. **(E)** A heat map demonstrating the expression levels of the commonly downregulated genes of *in vivo* and *in vitro* SARS-CoV-2 infection–related genes that were shared with severe ARDS-related genes. **(F)** A bar chart demonstrating the enriched gene ontology for the commonly upregulated or downregulated genes between *in vivo* and *in vitro* SARS-CoV-2 infection. **(G)** A bar chart demonstrating the enriched gene ontology for the contra-regulated genes between *in vivo* and *in vitro* COVID-19 infection. **(H)** A schematic graph demonstrating the balance of the virus promoting and defense genes of the SARS-CoV-2-infected epithelial cells.

Due to the limitation of the number of patients that were involved in each data set, we tried to overlap the data obtained from both *in vitro* and *in vivo* studies and found 132 intersecting genes among the four data sets in the current study (Figure 5A). Comparing the shared genes of *in vivo* and *in vitro* SARS-CoV-2-infection-related genes, both commonly regulated and contra-regulated genes were found (Figure 5B). Seventy-one commonly regulated genes were shared with the severity genes, 10 upregulated and 61 downregulated, and 61 were contra-regulated (Figures 5C–E and Supplementary Figure 7A). Gene enrichment analysis showed that the commonly regulated genes of *in vivo* and *in vitro* SARS-CoV-2 infection were classified to the cell activation and immune responses (Figure 5F), whereas the contra-regulated genes belonged to the intracellular organization (Figure 5G). Combined with the gene expression information obtained from *in vivo* studies, the *in vitro* gene expression data were able to demonstrate the virus promoting and inhibiting genes that related to disease severity and potential outcome of COVID-19 patients (Figure 5H and Supplementary Figure 7B).

## Discussion

In the current analysis, several data sets of single-cell RNA sequencing were adopted to study the gene expression in the BALF cells in response to SARS-CoV-2 infection. Correlations between viral and host transcriptomes from single cells allowed us to interrogate the gene signatures of infected compared with uninfected bystander cells. Although epithelial cells have a low viral transcript load, and all major immune cell types show high infection rates, only epithelial cells show virus-specific responses. Comparing the SARS-CoV-2-infection and the severe ARDS-related genes in the epithelial cells, we revealed possible targets within SARS-CoV-2-infected epithelial cells and were able to enrich the relevant signaling pathways involved.

During the treatment of a viral infection, there is generally a dilemma of treatment timing because the virus only multiplies at the early stages, thereby causing cell damage. In addition, in the later stages, with the enhancement of host immune functions, the virus tends to decrease, whereas the immune response at this time is usually very strong although these can result in death caused by COVID-19. From this, we can see that treatment at the early stages of the disease should focus on virus-infected target cells, and during SARS-CoV-2 infection, the critical target cells producing damage are epithelial cells, which is clearly reflected by the genetic analysis conducted here. Although epithelial cells do not have the most viral particles, they can exhibit the strongest immune response to viral particles. Thus, an early treatment should target epithelial cells. However, for epithelial cell therapy, there are two dilemmas. First, the replication of SARS-CoV-2 should be inhibited in the epithelial cells, and second, the antiviral innate immunity in these cells should be protected, possibly *via* the signaling pathway of IFN-inducible genes (Figure 2G). This therapeutic strategy should ensure that its targeted genes are only limited to epithelial cells rather than affecting immune cells and then affect the future immune response of the host to COVID-19.

In the present study, immune and epithelial cells were found to show high viral load heterogeneity although plasma cells maintained a low viral load state. Because immune cells lacked the SARS-CoV-2 viral receptor, ACE2, found on epithelial cells, the existence of other routes of infection has been suggested (Hoepel et al., 2021). Notably, a high viral load does not necessarily mean a low response. The data from this study indicate that the intracellular viral status could be compatible with a high viral response in epithelial cells. Epithelial cells could have an intracellular antiviral strategy, i.e., the activation of the type I IFN response, to balance the viral load (Steuerman et al., 2018). More importantly, a high viral load of epithelial cells does not necessarily imply a severe ARDS outcome or a poor convalescence. The current data demonstrate that many gene signatures of the high viral load fall into the mild group of ARDS. The simultaneous study on infected and bystander cells from COVID-19 patients with different severity or prognosis and on high-resolution alignment of gene expression variations could potentially convey more valuable information regarding host response pathways.

It is clear from previous reports that coronaviruses are highly sensitive to IFN treatment but use a variety of mechanisms to evade the intracellular induction of IFN (Lokugamage et al., 2020), which remain unclear. Although SARS-CoV-2 is adept at evading IFN induction in most cells, it appears to be poor at combating ISG. We demonstrate here that ISGs are highly sensitive to SARS-CoV-2 and that cells with high levels of the virus significantly increase the expression of some of the same ISGs. The effectiveness of how ISG and IFN induction are linked will facilitate virus elimination from these cells. The ability to link cellular functions with viral load status will also facilitate the future development of medical treatments and therapies for COVID-19 patients.

## Data Availability Statement

The datasets presented in this study can be found in online repositories. The names of the repository/repositories and accession number(s) can be found in the article/Supplementary Material.

## Author Contributions

LM, HQ, and JZ performed partial analysis and composed the manuscript. JSh, SH, QW, and ZW collected datasets and performed filtering for downstream analysis. SS edited the manuscript and brought strategic suggestions. JSo performed partial analysis, designed this study, supervised the whole study, and revised the manuscript. All authors contributed to the article and approved the submitted version.

## Conflict of Interest

The authors declare that the research was conducted in the absence of any commercial or financial relationships that could be construed as a potential conflict of interest. The handling editor declared a shared affiliation with one of the authors JSo at time of review.

## Publisher’s Note

All claims expressed in this article are solely those of the authors and do not necessarily represent those of their affiliated organizations, or those of the publisher, the editors and the reviewers. Any product that may be evaluated in this article, or claim that may be made by its manufacturer, is not guaranteed or endorsed by the publisher.

## Footnotes

1
https://satijalab.org/seurat/v3.1/integration.html

2
https://github.com/guokai8/VennDetail

3
https://github.com/taiyun/corrplot/

4
https://string-db.org/

5
http://COVID19.cancer-pku.cn/

## Supplementary Material

Supplementary Figure 1 Single cell RNA-seq analysis in the COVID-19 samples. **(A)** A UMAP diagram showing the 10 main cell types in the BALF samples. **(B)** A UMAP diagram showing the overlay of the main cell types of the COVID-19–related pneumonia (covid19), control (npnm), and bacterial pneumonia (pnm) samples. **(C)** A UMAP diagram showing the main cell clusters in the SARS-CoV-2–infected cells. **(D)** Violin plots showing the expression of COVID-19–related genes in the main BALF cell types.

Supplementary Figure 2 Shared DEGs in the COVID-19–related myeloid, epithelial, and endothelial cells. **(A)** Dot plots demonstrating the expression patterns and levels of the shared DEGs of myeloid, epithelial, and endothelial cells in the SARS-CoV-2 positive and negative cells. **(B)** Dot plots demonstrating the expression patterns and levels of the shared DEGs of myeloid, epithelial, and endothelial cells in the COVID-19–related pneumonia (covid19), control (npnm), and bacterial pneumonia (pnm) samples.

Supplementary Figure 3 Transcriptome profiling of SARS-CoV-2 heavy loaded cells. **(A)** Violin plots demonstrating the expression patterns and levels of the COVID-19–related genes in SARS-CoV-2–infected and non-infected cells. **(B)** Violin plots demonstrating the expression patterns and levels of the COVID-19–related genes in the main cell types.

Supplementary Figure 4 Relationship between COVID-19-related genes and DEGs. **(A)** Feature plots revealing the relationship between COVID-19-related N and ORF1ab gene expression in the main cell types and **(B)** in heavily loaded SARS-CoV-2 cells. **(C)** A heat map revealing the relationship between COVID-19–related genes and DEGs in heavily loaded SARS-CoV-2 monocytes. **(D)** A heat map revealing the relationship between COVID-19–related genes and DEGs in heavily loaded SARS-CoV-2 macrophages. **(E)** A heat map revealing the relationship between COVID-19–related genes and DEGs in heavily loaded SARS-CoV-2 CD8 + T cells. **(F)** A heat map revealing the relationship between COVID-19–related genes and DEGs in heavily loaded SARS-CoV-2 neutrophils. **(G)** A heat map revealing the relationship between COVID-19–related genes and DEGs in heavily loaded SARS-CoV-2 CD4 + T cells. **(H)** The PPI network demonstrating the COVID-19–related infection-correlated hub gene signatures in the SARS-CoV-2–infected epithelial cells. The molecular complex detection (MCODE) system was used to highlight the main network-connected genes.

Supplementary Figure 5 Shared DEGs in the non-infected cells compared with bacterial pneumonia and control cells. **(A)** A volcano plot demonstrating the fold change and the significance of the DEGs in epithelial cells from severe and mild ARDS BALF samples. **(B)** The shared gene signatures between SARS-CoV-2 infection-correlated genes and the severe ARDS-related genes. The PPI network demonstrates the shared gene signatures between SARS-CoV-2 infection-correlated genes and the severe ARDS-related genes. The oval shaped structures indicate the upregulated genes, and the rectangles indicate the downregulated genes in severe ARDS. Genes labeled with light blue text were positively correlated with the SARS-CoV-2 infection.

Supplementary Figure 6 Gene expression in SARS-CoV-2–infected epithelial cell lines. **(A)** Violin plots demonstrating the expression patterns and levels of the *in vivo* SARS-CoV-2 infection-related PPI hub genes in the SARS-CoV-2–infected epithelial cell lines. **(B)** Feature plots revealing the relationship between COVID-19-related genes and *in vivo* SARS-CoV-2 infection-related PPI hub genes in the SARS-CoV-2–infected epithelial cell lines.

Supplementary Figure 7 Overlapping of *in vivo* and *in vitro* gene expression and disease severity. **(A)** A heat map demonstrating the expression levels of the contra-regulated genes of *in vivo* and *in vitro* SARS-CoV-2 infection-related genes that were shared with genes occurring in severe ARDS. **(B)** A flow chart demonstrating the analysis strategy of screening the virus-promoting and defense genes by combining the information from *in vivo* and *in vitro* gene expression data and the disease severity.

## Abbreviations

COVID-19: coronavirus disease 2019
SARS-CoV-2: severe acute respiratory syndrome coronavirus 2
ARDS: acute respiratory distress syndrome
DEG: differentially expressed gene
BALF: bronchoalveolar lavage fluid
PCA: principal component analysis
UMAP: uniform manifold approximation and projection
GSEA: Gene Set Enrichment Analysis
PPI: protein-protein interaction
VEGs: virally expressed genes
nHTBE: normal human tracheal bronchial epithelial
ISGs: interferon induced genes.
