# Supplementary figures and images for: Transcriptomic Signatures of Airway Epithelium Infected With SARS-CoV-2: A Balance Between Anti-infection and Virus Load
Source: Front Cell Dev Biol. 2021 Aug 23;9:735307. doi: 10.3389/fcell.2021.735307 (PMC8419361; doi:10.3389/fcell.2021.735307)

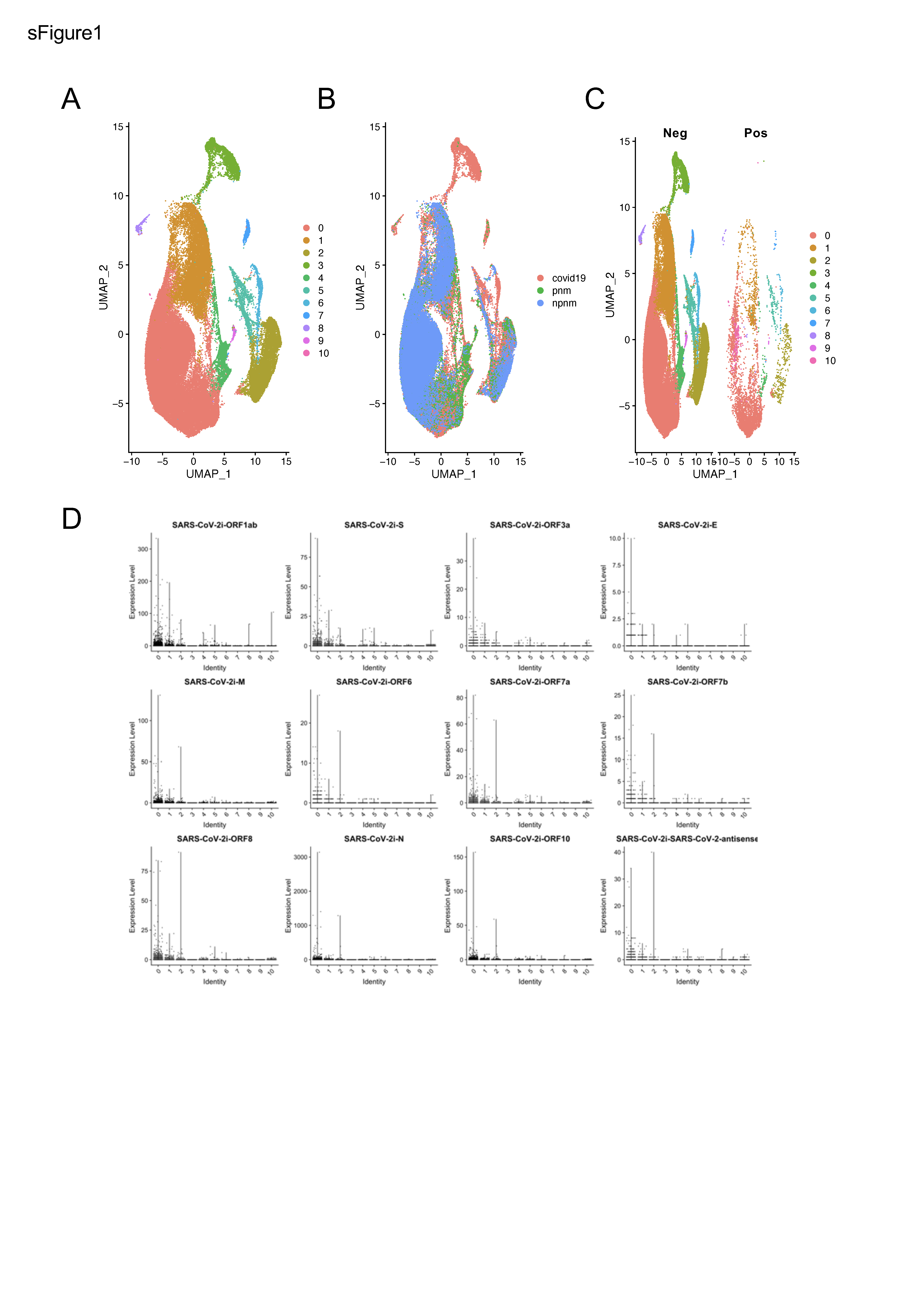

Supplement: Supplementary Figure 1 — Single cell RNA-seq analysis in the COVID-19 samples. (A) A UMAP diagram showing the 10 main cell types in the BALF samples. (B) A UMAP diagram showing the overlay of the main cell types of the COVID-19–related pneumonia (covid19), control (npnm), and bacterial pneumonia (pnm) samples. (C) A UMAP diagram showing the main cell clusters in the SARS-CoV-2–infected cells. (D) Violin plots showing the expression of COVID-19–related genes in the main BALF cell types. [file Image_1.TIFF]

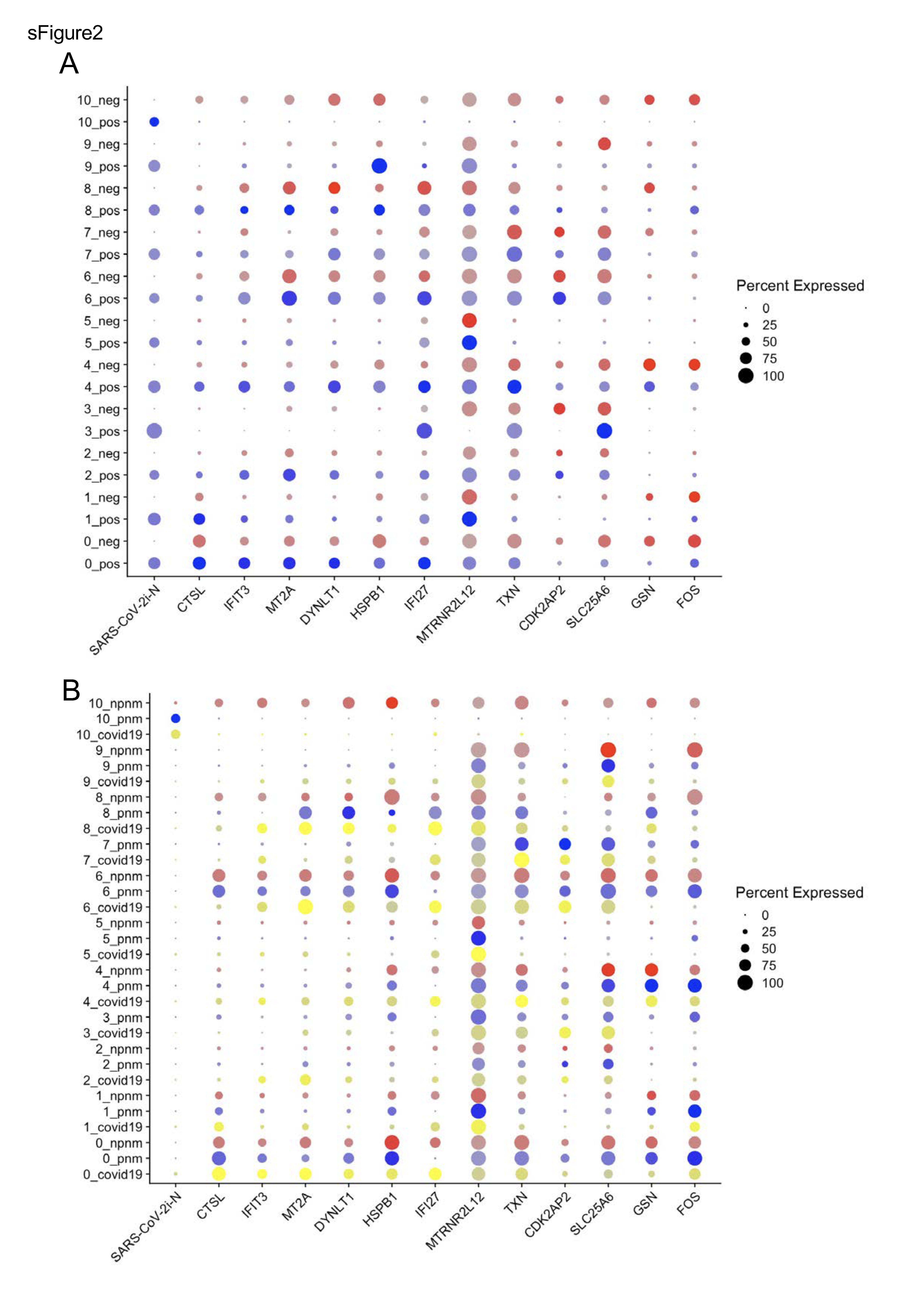

Supplement: Supplementary Figure 2 — Shared DEGs in the COVID-19–related myeloid, epithelial, and endothelial cells. (A) Dot plots demonstrating the expression patterns and levels of the shared DEGs of myeloid, epithelial, and endothelial cells in the SARS-CoV-2 positive and negative cells. (B) Dot plots demonstrating the expression patterns and levels of the shared DEGs of myeloid, epithelial, and endothelial cells in the COVID-19–related pneumonia (covid19), control (npnm), and bacterial pneumonia (pnm) samples. [file Image_2.TIFF]

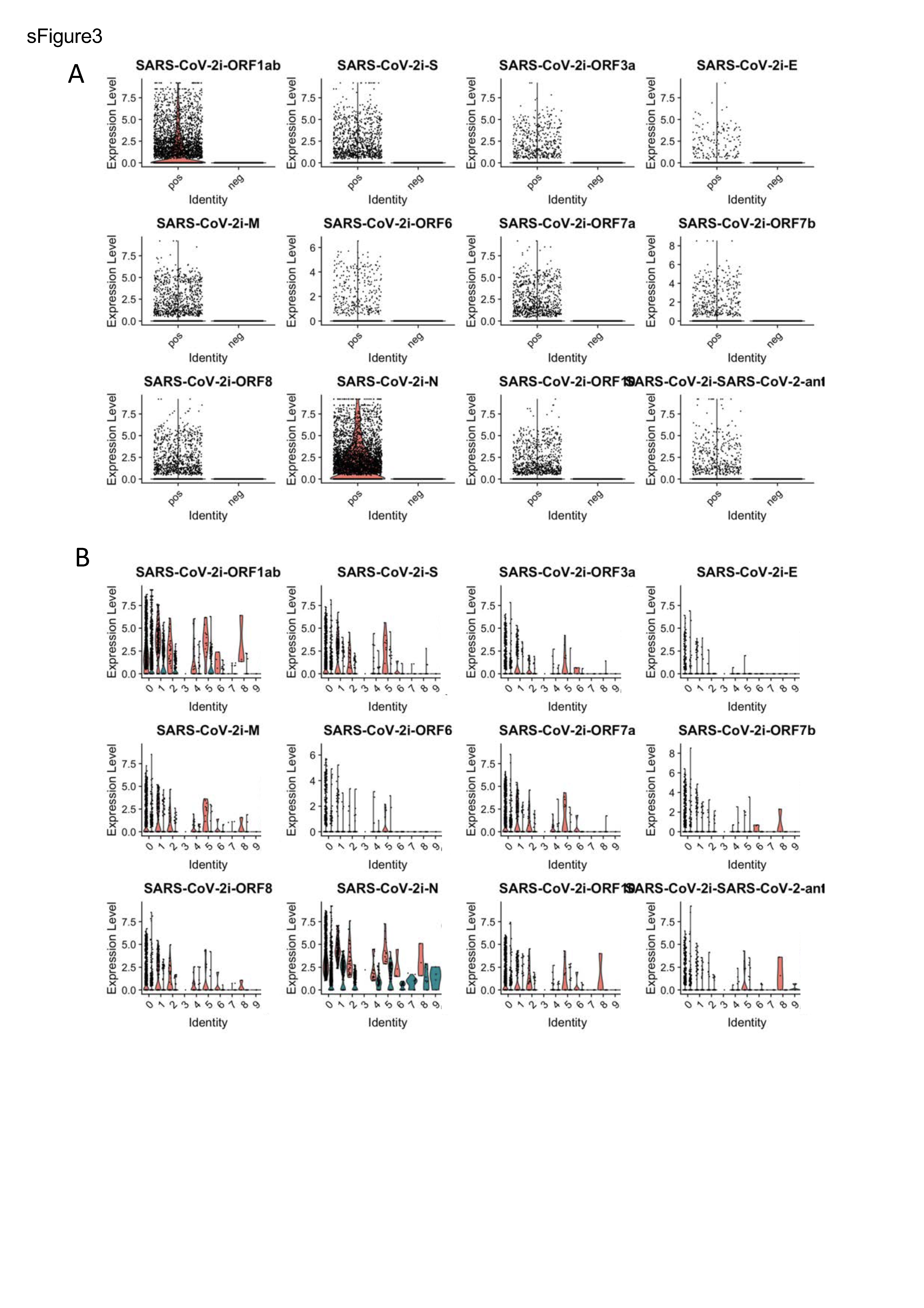

Supplement: Supplementary Figure 3 — Transcriptome profiling of SARS-CoV-2 heavy loaded cells. (A) Violin plots demonstrating the expression patterns and levels of the COVID-19–related genes in SARS-CoV-2–infected and non-infected cells. (B) Violin plots demonstrating the expression patterns and levels of the COVID-19–related genes in the main cell types. [file Image_3.TIFF]

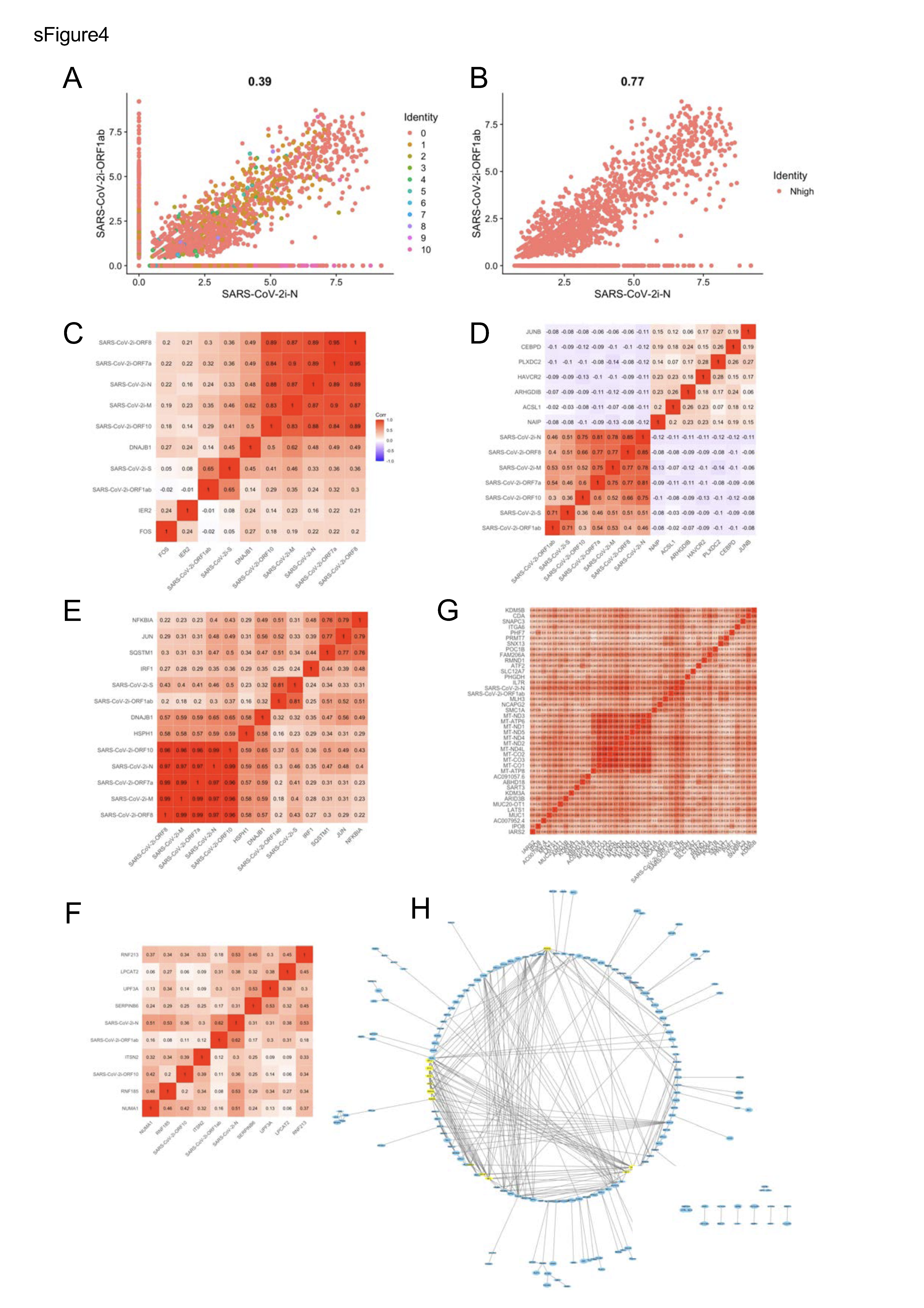

Supplement: Supplementary Figure 4 — Relationship between COVID-19-related genes and DEGs. (A) Feature plots revealing the relationship between COVID-19-related N and ORF1ab gene expression in the main cell types and (B) in heavily loaded SARS-CoV-2 cells. (C) A heat map revealing the relationship between COVID-19–related genes and DEGs in heavily loaded SARS-CoV-2 monocytes. (D) A heat map revealing the relationship between COVID-19–related genes and DEGs in heavily loaded SARS-CoV-2 macrophages. (E) A heat map revealing the relationship between COVID-19–related genes and DEGs in heavily loaded SARS-CoV-2 CD8 + T cells. (F) A heat map revealing the relationship between COVID-19–related genes and DEGs in heavily loaded SARS-CoV-2 neutrophils. (G) A heat map revealing the relationship between COVID-19–related genes and DEGs in heavily loaded SARS-CoV-2 CD4 + T cells. (H) The PPI network demonstrating the COVID-19–related infection-correlated hub gene signatures in the SARS-CoV-2–infected epithelial cells. The molecular complex detection (MCODE) system was used to highlight the main network-connected genes. [file Image_4.TIFF]

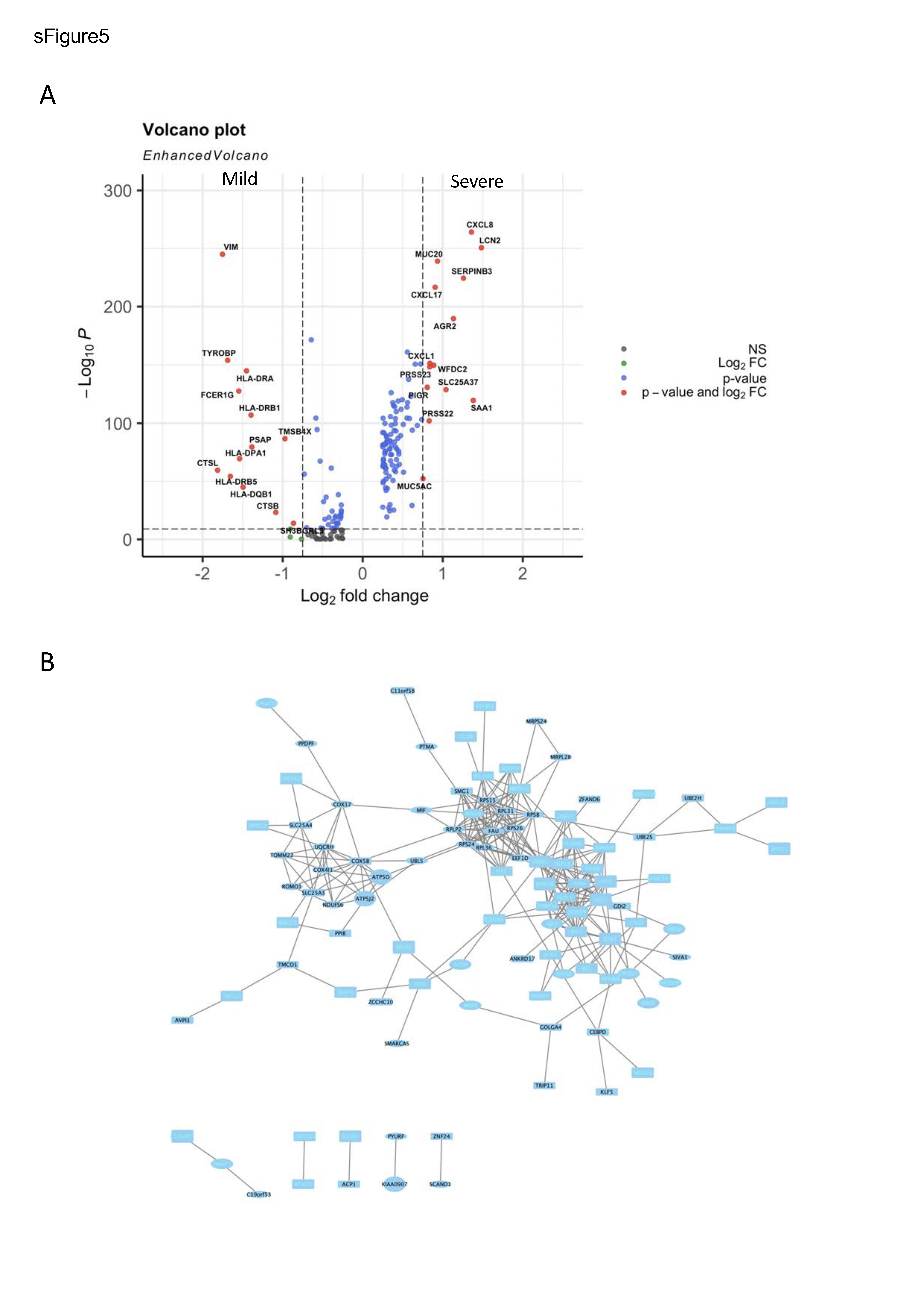

Supplement: Supplementary Figure 5 — Shared DEGs in the non-infected cells compared with bacterial pneumonia and control cells. (A) A volcano plot demonstrating the fold change and the significance of the DEGs in epithelial cells from severe and mild ARDS BALF samples. (B) The shared gene signatures between SARS-CoV-2 infection-correlated genes and the severe ARDS-related genes. The PPI network demonstrates the shared gene signatures between SARS-CoV-2 infection-correlated genes and the severe ARDS-related genes. The oval shaped structures indicate the upregulated genes, and the rectangles indicate the downregulated genes in severe ARDS. Genes labeled with light blue text were positively correlated with the SARS-CoV-2 infection. [file Image_5.TIFF]

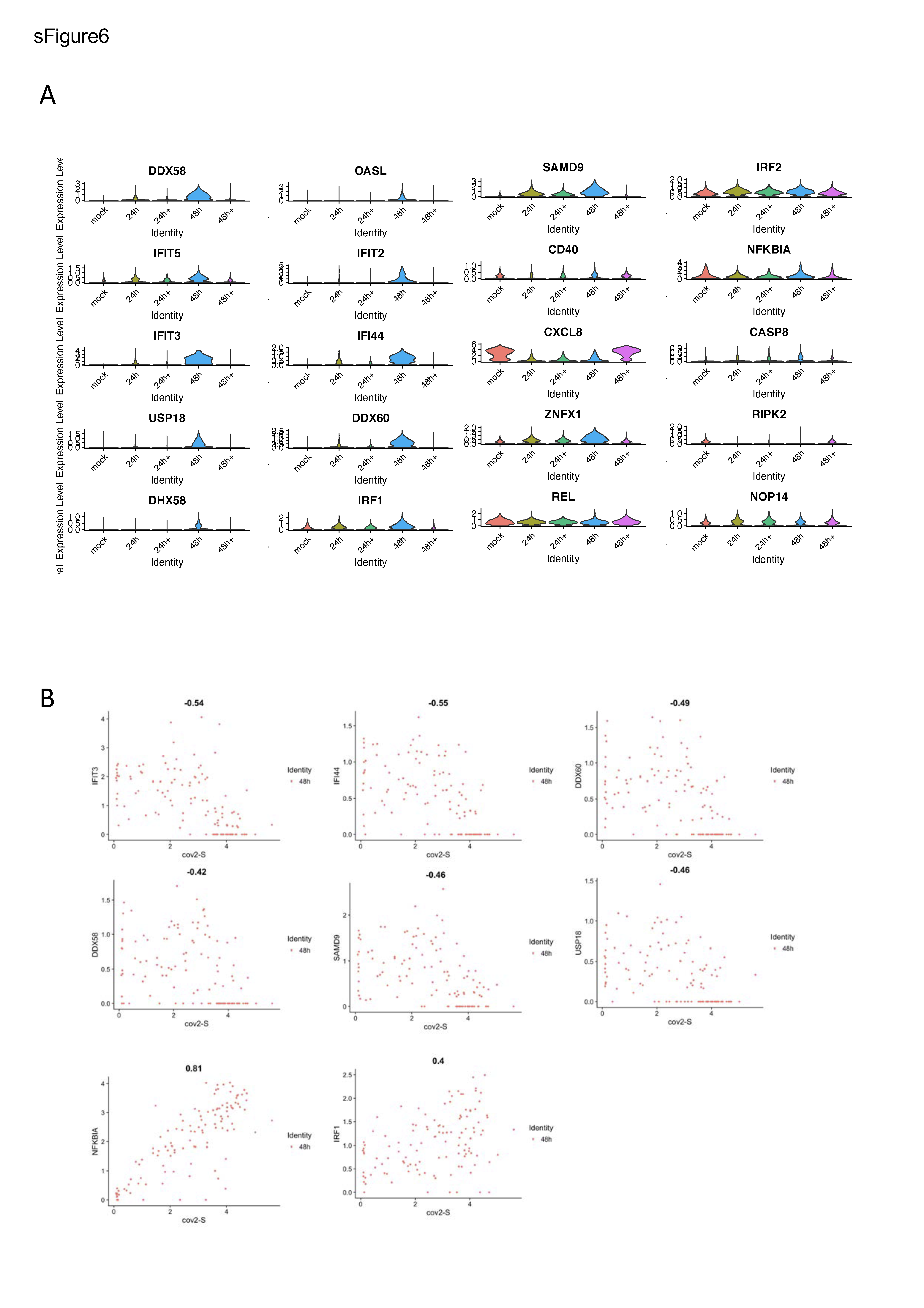

Supplement: Supplementary Figure 6 — Gene expression in SARS-CoV-2–infected epithelial cell lines. (A) Violin plots demonstrating the expression patterns and levels of the in vivo SARS-CoV-2 infection-related PPI hub genes in the SARS-CoV-2–infected epithelial cell lines. (B) Feature plots revealing the relationship between COVID-19-related genes and in vivo SARS-CoV-2 infection-related PPI hub genes in the SARS-CoV-2–infected epithelial cell lines. [file Image_6.TIFF]

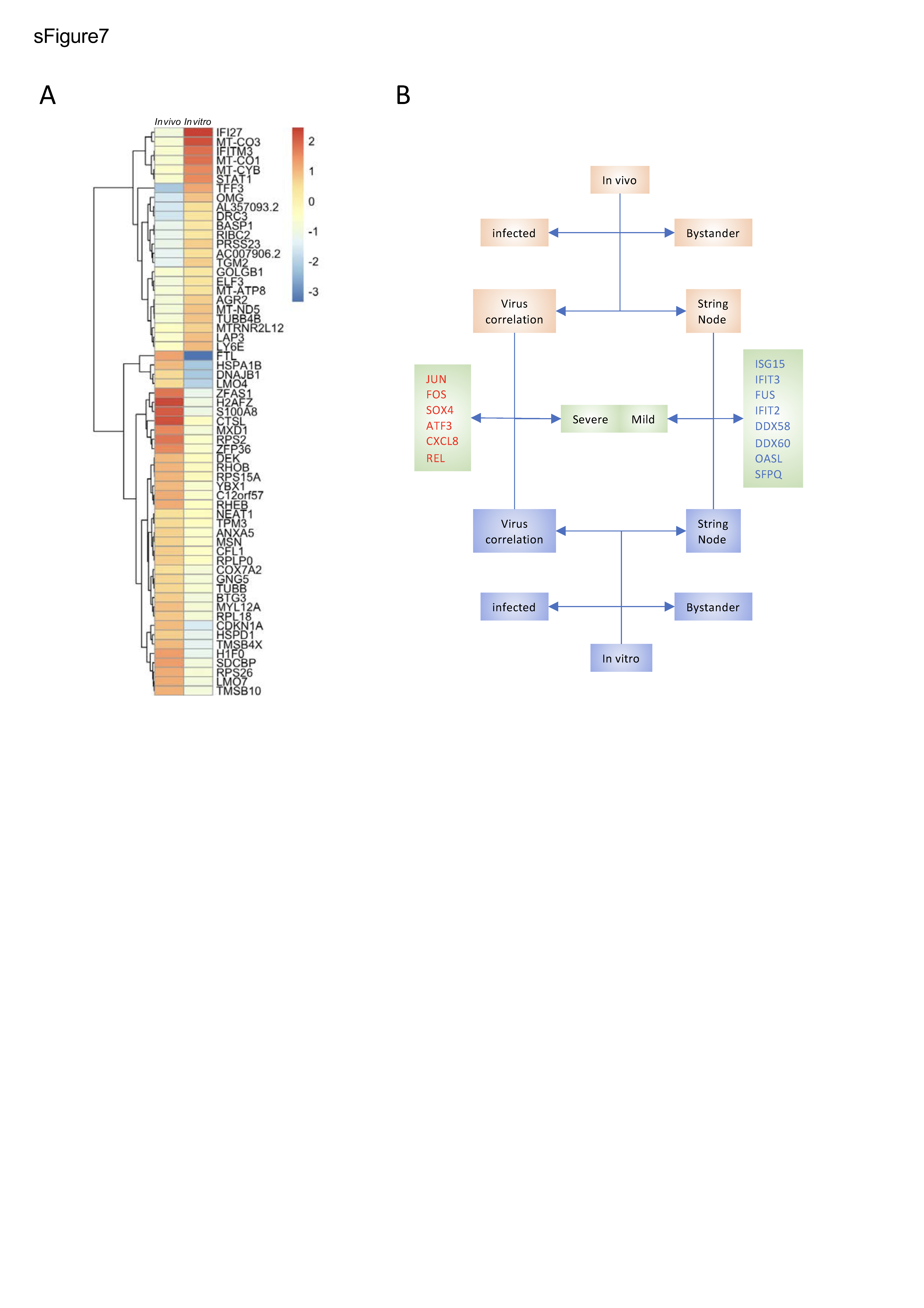

Supplement: Supplementary Figure 7 — Overlapping of in vivo and in vitro gene expression and disease severity. (A) A heat map demonstrating the expression levels of the contra-regulated genes of in vivo and in vitro SARS-CoV-2 infection-related genes that were shared with genes occurring in severe ARDS. (B) A flow chart demonstrating the analysis strategy of screening the virus-promoting and defense genes by combining the information from in vivo and in vitro gene expression data and the disease severity. [file Image_7.TIFF]
